# Microbiota-derived short-chain fatty acids in hematopoietic stem cell transplantation: immunomodulation at the host-microbiota interface

**DOI:** 10.3389/fmicb.2026.1754099

**Published:** 2026-02-06

**Authors:** Crystel Hajjar, Ed J. Kuijper, Marie-José Butel, Gaelle Khoury, May Mallah, Dolla Karam Sarkis, Philippe Lesnik, Wilfried Le Goff, Ali Bazarbachi, Marianne Abifadel

**Affiliations:** 1 Laboratory of Microbiology, Faculty of Pharmacy, Pôle Technologie-Santé, Saint Joseph University of Beirut, Beirut, Lebanon; 2 National Expertise Center for Clostridioides Difficile Infections, Leiden University Center for Infectious Diseases, Leiden, Netherlands; 3 National Institute for Public Health and the Environment, Bilthoven, Netherlands; 4 INSERM, UMR-S 1139, Physiopathologie et Pharmacotoxicologie Placentaire Humaine Microbiote Pré- et Postnatal (3PHM), Université Paris Cité, Paris, France; 5 INSERM, UMR-S 1166, Unité de Recherche sur les Maladies Cardiovasculaires et Métaboliques (ICAN), Sorbonne Université, Paris, France; 6 Bone Marrow Transplantation Program, Department of Internal Medicine, American University of Beirut Medical Center, Beirut, Lebanon; 7 Laboratory of Biochemistry and Molecular Therapeutics, Faculty of Pharmacy, Pôle Technologie-Santé, Saint Joseph University of Beirut, Beirut, Lebanon

**Keywords:** butyrate, epigenetic regulation, graft-vs.-host disease (GvHD), gut microbiota, gut-immune axis, hematopoietic stem cell transplantation (HSCT), intestinal barrier, short-chain fatty acids (SCFAs)

## Abstract

Hematopoietic stem cell transplantation (HSCT) remains a cornerstone treatment for many hematological malignancies, but its clinical success is still challenged by graft-vs.-host disease (GvHD), infectious complications, and the profound microbial disruptions caused by conditioning, antibiotics, and hospitalization. Over the past few years, a growing body of work has highlighted how tightly post-transplant immunity is linked to the state of the gut microbiota. In particular, short-chain fatty acids (SCFAs), especially butyrate, have emerged as key microbial metabolites involved in maintaining epithelial barrier function, moderating inflammatory responses, and supporting regulatory T-cell homeostasis. In this review, we bring together current evidence on the SCFA-gut-immune axis in the setting of HSCT, with a focus on how transplant-related dysbiosis alters SCFA availability and contributes to immune imbalance. We also discuss the potential of strategies designed to restore or enhance SCFA production, ranging from dietary fiber interventions to next-generation probiotics and other microbiota-directed approaches. Overall, by better understanding and eventually harnessing the metabolic capacity of the gut microbiota, SCFA-centered therapies may offer new opportunities to support immune recovery, reduce GvHD risk, and improve outcomes for HSCT recipients. Still, well-designed clinical trials are needed to determine how these approaches can be safely and effectively integrated into transplant care.

## Graphical Abstract

**Figure:**
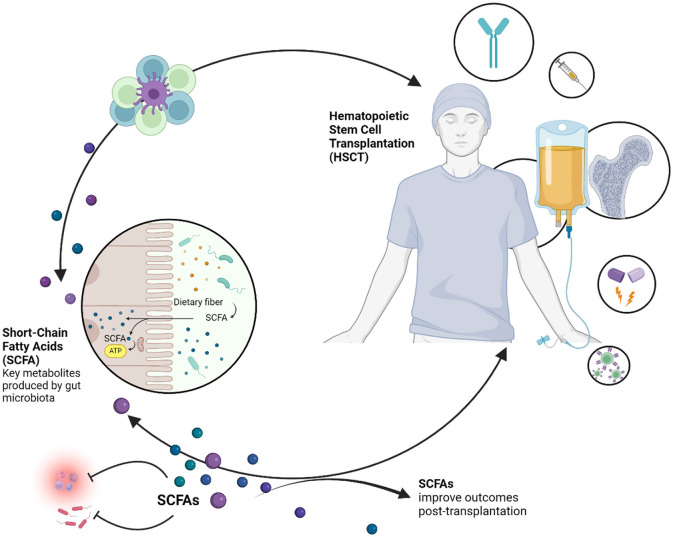

## Introduction

Hematopoietic Stem Cell Transplantation (HSCT) is a lifesaving procedure for patients with hematological malignancies (Trunk et al., 2024). However, HSCT exerts profound and often detrimental effects on the gut microbiota. Throughout the pre-, peri-, and post-transplant phases, patients are exposed to various interventions, such as chemotherapy, radiotherapy, broad-spectrum antibiotics, and immunosuppressive therapies, that profoundly alter the gut microbial landscape (Habibi and Rashidi, 2023). This disruption leads to reduced microbial diversity and a marked depletion of beneficial short-chain fatty acid (SCFA)-producing bacteria, which are essential for maintaining gastrointestinal integrity and immune homeostasis (Habibi and Rashidi, 2023).

Microbiota injury is particularly pronounced in recipients of allogeneic HSCT, especially those who develop graft-vs.-host disease (GvHD), a major cause of morbidity and mortality following transplantation. While the broader influence of the gut microbiome on HSCT outcomes has been increasingly recognized, the specific roles and mechanisms of SCFAs in this context remain insufficiently explored (Yue et al., 2024). Although prior studies have reported alterations in microbial composition and metabolite profiles after HSCT, a focused synthesis of SCFA-mediated immunomodulatory pathways and their therapeutic potential is lacking (Riwes and Reddy, 2020).

Emerging evidence suggests that reduced SCFA levels correlate with increased immune dysregulation and more severe manifestations of GvHD (Yue et al., 2024). These observations highlight the need to better understand the SCFA-gut-immune axis in the transplant setting and to explore avenues for clinical translation.

This narrative review consolidates current evidence on SCFAs in HSCT and provides a critical perspective on their potential as therapeutic allies (Azhar Ud Din et al., 2025; Song et al., 2024). We examine:

- How HSCT alters gut microbial ecology and reduces SCFA-producing taxa;
- The immunomodulatory effects of SCFAs, including their roles in regulatory T cell induction, intestinal barrier function, and inflammation control;
- SCFA-based therapeutic strategies, ranging from diet and prebiotics to probiotics and microbial consortia, for preventing or mitigating GvHD and improving post-transplant recovery;
- And finally, we discuss future directions for integrating SCFA modulation into personalized medicine approaches in HSCT recipients.

## Methodology

### Search strategy and databases

This narrative review was based on a structured literature search designed to comprehensively evaluate the role of SCFAs in HSCT, with an emphasis on their immunomodulatory effects and therapeutic potential. Relevant literature was identified using PubMed and Google Scholar, covering recent articles, theses, and books.

Search terms included combinations of the following keywords: “Hematopoietic Stem Cell Transplantation (HSCT),” “Short-chain fatty acids (SCFAs),” “Gut microbiome,” “Graft-vs.-host disease (GvHD),” “Immune modulation,” and “Microbiota-directed therapy.” Boolean operators (AND, OR) were used to refine results. Manual backward and forward citation tracking of key review articles and original studies was performed to identify additional eligible sources.

### Inclusion and exclusion criteria for summary tables

Studies were included in the summary tables if they met any of the following criteria:

- Investigated the role of SCFAs in HSCT recipients;
- Evaluated SCFA-targeted interventions (e.g., dietary fiber, probiotics, fecal microbiota transplantation) in clinical or preclinical HSCT contexts.

Studies were excluded if they:

- Focused on microbiome pathways unrelated to SCFAs;
- Were non-peer-reviewed sources (e.g., conference abstracts, commentaries);
- Did not report mechanistic or outcome-based data on SCFA activity in immune modulation or HSCT-related complications.

For each included study, the following data were extracted:

- SCFA levels and gut microbiota composition post-HSCT;
- Immunomodulatory effects of SCFAs on GvHD, immune reconstitution, and inflammatory pathways;
- SCFA-based therapeutic strategies involving prebiotics, probiotics, dietary interventions, or engineered microbiota.

### Quality assessment and limitations

As a narrative review, no formal risk of bias assessment was conducted. However, studies were critically appraised based on:

- Study design (preclinical models, clinical trials, or observational cohorts);
- Direct relevance to SCFA metabolism, production, and immune modulation in the context of HSCT;
- Mechanistic clarity and translational potential of SCFA-driven pathways.

While this approach allows for conceptual synthesis across diverse study types, it inherently carries certain limitations:

- Risk of selection bias: Although a structured search was performed, study selection was guided by author discretion based on scientific relevance and mechanistic insight.
- Narrative synthesis limitations: The absence of meta-analytic integration precludes quantitative effect size estimation or ranking of therapeutic efficacy.
- Heterogeneity in included studies: Substantial variability exists in SCFA measurement techniques, intervention modalities, and outcome definitions across studies.
- Publication bias: Positive or preclinical findings are more likely to be published and may overrepresent the apparent benefit of SCFAs.
- Language and accessibility bias: Only English-language, publicly accessible sources were considered.

Despite these constraints, efforts were made to ensure breadth and depth of coverage, focusing on mechanistic insights and translational relevance, which are particularly valuable for hypothesis generation in this emerging field.

## Hematopoietic stem cell transplantation: a lifesaving therapy with immunological risks

HSCT has emerged as a curative therapy for a range of hematological malignancies, bone marrow disorders, and inherited immune deficiencies (Riwes and Reddy, 2020; Pitea et al., 2023). This procedure involves intravenous infusion of hematopoietic stem cells obtained either from the patient (autologous) or a compatible donor (allogeneic) to reconstitute the recipient's immune system and blood-forming capacity (Khalil and Maher, 2024). While HSCT offers the potential for durable remission and long-term survival, it remains burdened by serious complications.

**Figure 1:**
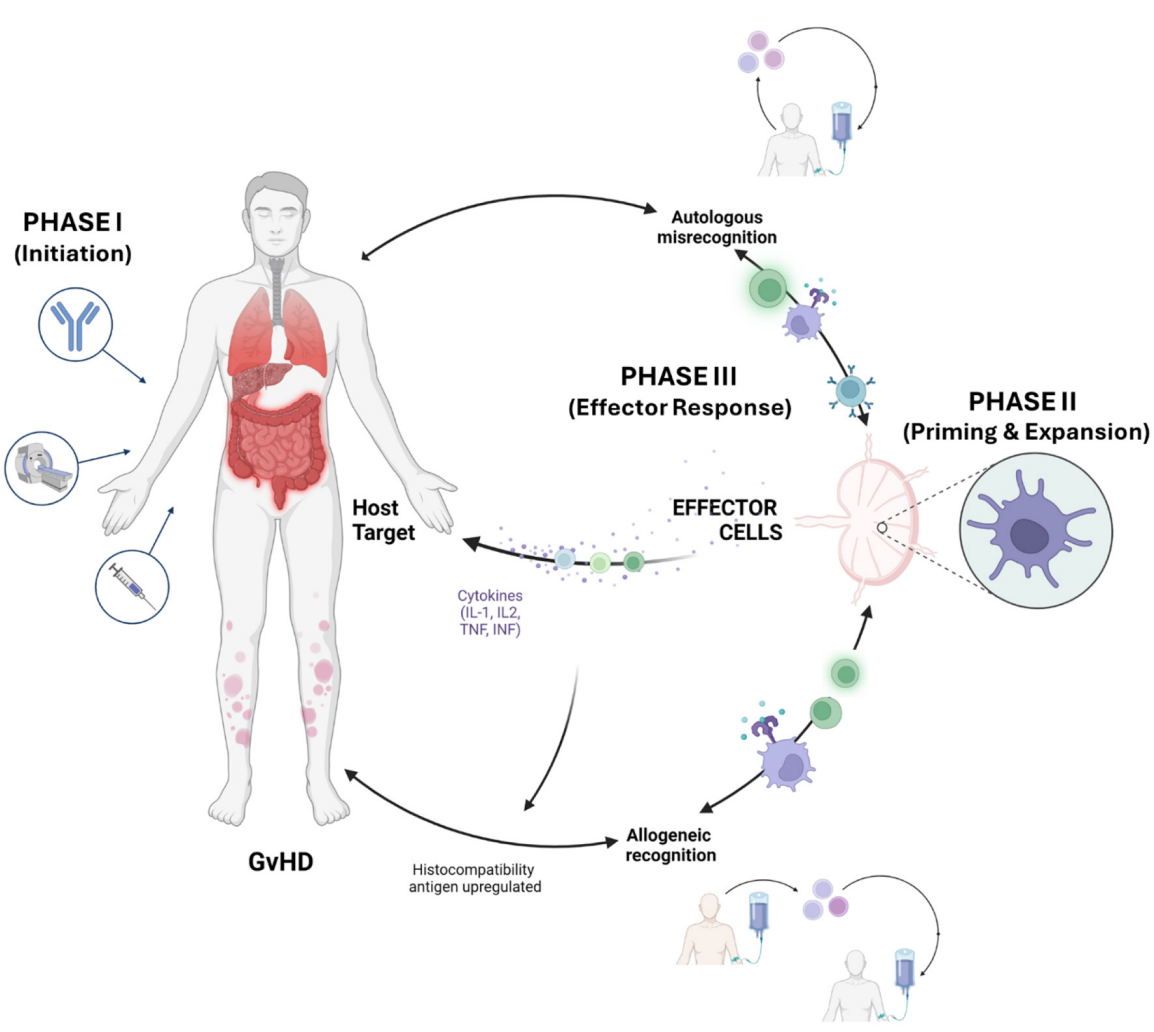
Schematic of the Three-Phase Pathophysiology of Graft-vs.-host disease. The development of Graft-vs.-host disease (GvHD) progresses through three distinct stages: (Phase I) Initiation of host tissue damage and cytokine release by conditioning regimens; (Phase II) Priming and clonal expansion of alloreactive donor T cells; and (Phase III) The effector phase characterized by autologous misrecognition, where activated donor T cells infiltrate and destroy host target tissues (skin, GI tract, liver, and lungs). Microbial metabolites, such as SCFAs, are critical modulators across this timeline, influencing both the initial barrier damage in Phase I and the subsequent T cell polarization in Phases II and III.

A principal challenge is the development of GvHD (Li et al., 2022), a potentially life-threatening condition that arises when immunocompetent donor T cells mount an alloreactive response against recipient tissues (Wang et al., 2015; Heidegger et al., 2014). GvHD may present in acute or chronic forms, with clinical manifestations affecting multiple organs including the skin, gastrointestinal tract, liver, and lungs (Vaillant et al., 2024) (Figure 1). The severity of GvHD is a critical determinant of HSCT success and patient survival (Faraci et al., 2024).

Acute GvHD (aGvHD) occurs in approximately 35–50% of allogeneic HSCT recipients, with associated mortality rates ranging from 15–20% (Bu et al., 2023). Chronic GvHD (cGvHD), which develops in 30–70% of patients, contributes significantly to long-term morbidity and is associated with mortality rates of 20–30% (Teshima et al., 2016). Risk factors influencing GvHD severity include human leukocyte antigen (HLA) disparity, conditioning intensity, and preexisting comorbidities (Dulery, 2023).

Clinically, aGvHD often presents with erythematous skin rash, gastrointestinal disturbances such as diarrhea and abdominal pain, and hepatic injury with jaundice (Malard et al., 2023a). cGvHD, in contrast, is characterized by immune-mediated tissue fibrosis and can resemble autoimmune disease, manifesting as scleroderma-like skin changes, sicca symptoms, hepatic fibrosis, and chronic pulmonary complications such as bronchiolitis obliterans (Hamilton, 2021). These complications markedly impair quality of life and may lead to irreversible organ damage, thereby impacting long-term prognosis (Vaillant et al., 2024).

The development of GvHD is classically described as a three-phase process (Zeiser and Blazar, 2017; Flowers and Martin, 2015; Jagasia et al., 2015) (Figure 1): Phase I (The Activation Phase) is initiated by the pre-transplant conditioning regimen, which damages recipient tissues and triggers the release of inflammatory cytokines and the upregulation of host histocompatibility antigens (Adams, 2024). This pro-inflammatory environment facilitates Phase II (The Expansion and Differentiation Phase), in which donor T cells recognize these host antigens as foreign. This recognition leads to the rapid clonal expansion and differentiation of donor cells into effector T cells, a process further amplified by a “cytokine storm” involving IL-2, TNF-α, and IFN-γ (Miura et al., 2002). Finally, Phase III (The Effector Phase) occurs when these activated donor cells migrate to and attack host target organs. This stage represents a state of autologous misrecognition, where the donor immune system, while technically recognizing foreign alloantigens, erroneously targets the host's healthy epithelial and stromal tissues as if they were malignant or infected, leading to the clinical manifestations of GvHD (Ferrara et al., 2009).

## The gut microbiome in HSCT: a central modulator of transplant outcomes

The human gut hosts a complex and diverse community of microorganisms, including bacteria, archaea, and fungi, collectively referred to as the gut microbiota (Minagar and Jabbour, 2025). Strictly anaerobic bacteria constitute the majority of this ecosystem, deriving energy primarily through fermentation of dietary fibers and host-derived substrates, and producing key metabolic by-products such as SCFAs, carbon dioxide, hydrogen, and methane (Ríos-Covián et al., 2016). This microbial community plays a key role in host physiology by supporting nutrient metabolism, immune system maturation, and resistance to pathogen colonization (Pianko and Golob, 2022).

Among its most critical functions is the regulation of host immunity via complex, bidirectional interactions with intestinal epithelial and immune cells (Di Vincenzo et al., 2024). These dynamic microbiota-immune crosstalk mechanisms help preserve intestinal homeostasis, prevent overgrowth of opportunistic microbes, and reinforce the epithelial barrier (Hammerhøj et al., 2024). Commensal microorganisms also provide protection by competing for nutrients and adhesion sites, producing antimicrobial compounds, and supporting mucosal integrity, all contributing to reduced infection risk and balanced immune activation (Di Vincenzo et al., 2024). Additionally, the gut microbiota delivers essential developmental signals to the host immune system and facilitates energy harvest from dietary components (Yoo et al., 2020).

HSCT, while curative for many hematological malignancies and immunodeficiencies (Trunk et al., 2024), induces profound disturbances to this delicate ecosystem. Conditioning regimens (including chemotherapy and radiotherapy), broad-spectrum antibiotics, immunosuppressive therapies, mucosal damage, and nutritional alterations collectively drive a collapse in microbial diversity and function (Romick-Rosendale et al., 2018; Montassier et al., 2015; Zhao et al., 2021; Fernandes et al., 2021). A clinical study of 119 HSCT recipients linked gut microbiota changes to an increased risk of neutropenic fever, suggesting that dysbiosis may contribute to immune dysfunction during post-transplant neutropenia (Schwabkey et al., 2022). A large multicenter study of 8,767 stool samples from 1,362 patients demonstrated that lower gut microbial diversity during the peri-engraftment phase was significantly associated with increased mortality (Peled et al., 2020). Complementing this, a prospective study in pediatric recipients found that higher pre-transplant microbial diversity was associated with markedly better overall survival (88.9% vs. 62.7%) and reduced incidence of aGvHD (20.0% vs. 44.4%) (Masetti et al., 2023).

Beyond reductions in alpha diversity, recent multi-omics analyses have illuminated key functional disruptions. In a prospective cohort, Artacho et al. (2024) observed the depletion of beneficial SCFA-producing *Clostridiales* and associated metabolites, including butyrate, propionate, and acetate, alongside expansion of *Staphylococcus* and *Enterococcus faecium*. Notably, GvHD severity was linked to reductions in microbial genes such as superoxide reductases, underscoring the impact of metabolic and immunological impairments beyond taxonomy alone.

**Figure 2:**
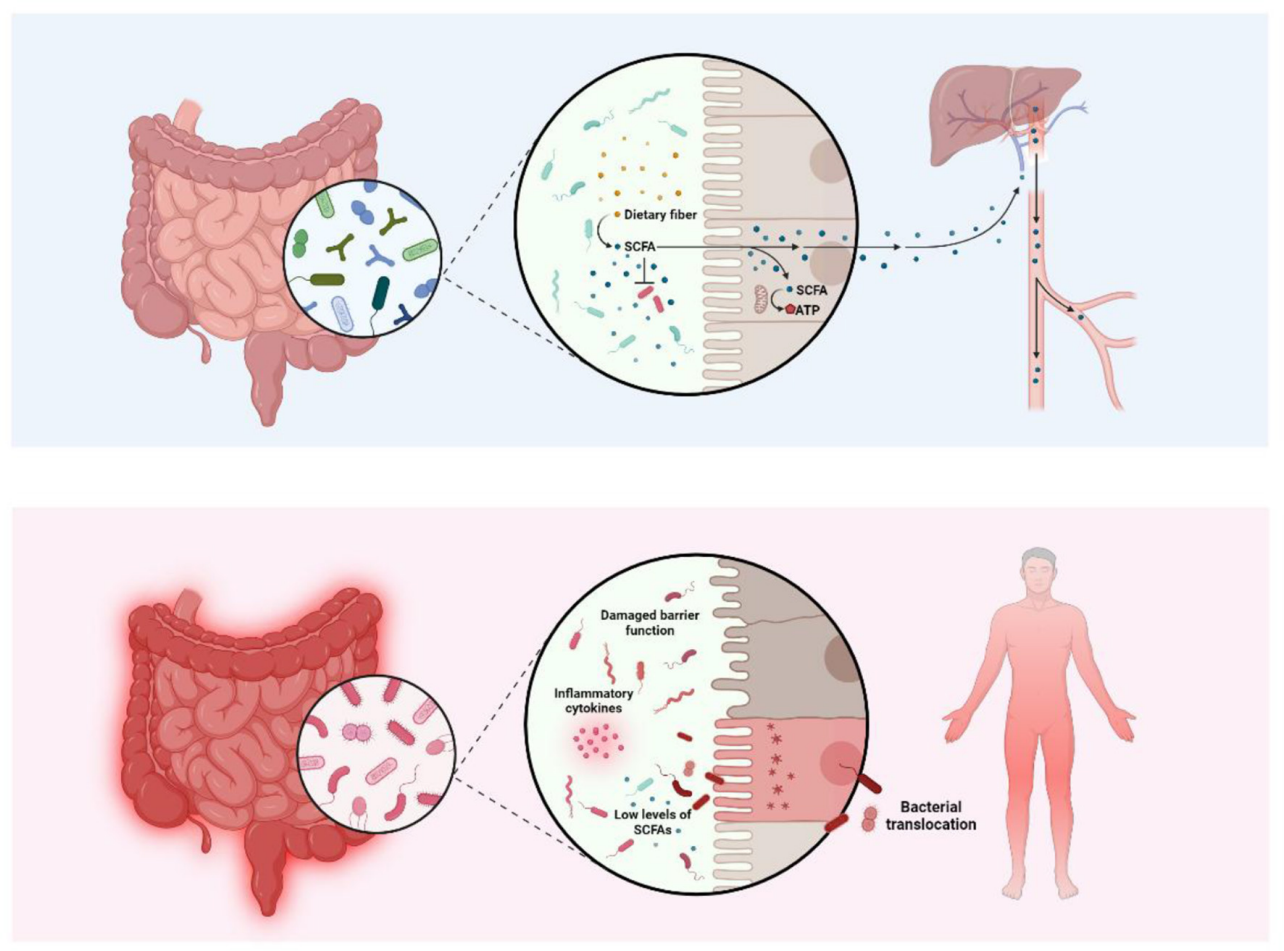
Gut microbiota balance and dysbiosis in the context of hematopoietic stem cell transplantation. The upper panel illustrates a healthy gut microbiota, in which commensal bacteria ferment dietary fiber into short-chain fatty acids (SCFAs). These metabolites are rapidly absorbed by colonocytes, serving as their primary energy source and reinforcing epithelial barrier integrity (Luu et al., 2020; Li et al., 2023). A portion of SCFAs enters the portal circulation, where they contribute to hepatic metabolic processes including lipogenesis and gluconeogenesis (Dalile et al., 2019). The lower panel depicts the post-hematopoietic stem cell transplantation (HSCT) gut, characterized by dysbiosis induced by conditioning regimens. This microbial disruption leads to a loss of SCFA-producing taxa, reduced SCFA availability, and compromised barrier function (Burgos da Silva et al., 2022). The ensuing epithelial damage facilitates bacterial translocation and triggers systemic inflammatory responses, including elevated cytokine production, which further exacerbates barrier breakdown and promotes graft-vs.-host disease (GvHD) (Ji et al., 2024; Peled et al., 2016; Rückert et al., 2022).

Colonization with multidrug-resistant organisms further exacerbates these disruptions. In a prospective study, Corcione et al. (2025) demonstrated that patients colonized with extended-spectrum β-lactamase (ESBL)-producing organisms exhibited distinct microbial profiles, with greater relative abundances of *Bifidobacterium, Clostridium, Blautia*, and *Akkermansia*. Persistent colonization was also associated with elevated rates of *Clostridioides difficile* infection and increased abundance of *Streptococcus*, a genus previously linked to aGvHD risk (Corcione et al., 2025). These findings highlight how exogenous microbial exposures modulate host-microbe dynamics and may influence post-transplant complications (Figure 2).

This HSCT-induced dysbiosis, marked by decreased microbial diversity, depletion of immunoregulatory taxa, and overrepresentation of pathobionts (Yoon and Yoon, 2018), results in a substantial reduction in critical SCFA producers such as *Clostridium, Ruminococcus, Blautia*, and *Faecalibacterium prausnitzii* (Romick-Rosendale et al., 2018; Peled et al., 2020; Burgos da Silva et al., 2022; Malard et al., 2018; Peled et al., 2018; Taur et al., 2018). One cohort study documented a significant increase in *Enterococcus* species post-transplant in patients who developed aGvHD (from 0.1% at baseline to 12.8%), with a concurrent decline in *Enterobacteriaceae* (Gavriilaki et al., 2024). Similarly, a systematic review of 10 studies comprising 490 pediatric HSCT recipients confirmed consistent reductions in microbial diversity and in SCFA-producing families, particularly *Ruminococcaceae*, among patients developing GvHD (Sohouli et al., 2025).

Importantly, beyond compositional changes, dysbiosis alters key host-metabolite signaling pathways. A prospective multi-omics study found that microbial disruption impairs bile acid metabolism, creating a feedback loop between intestinal microbes and immune cells that amplifies IL-1-mediated inflammation and worsens GvHD severity (Han et al., 2024).

Taken together, these studies suggest that the altered gut microbiota is not merely a bystander but a functional contributor to HSCT-associated complications, including GvHD, infections, mucositis, and graft failure. Disruption of epithelial barrier integrity, secondary to loss of commensals and expansion of proinflammatory taxa, may facilitate bacterial translocation, fueling systemic inflammation and immune dysregulation. While it remains unclear whether dysbiosis is a primary driver or a consequence of conditioning-induced immune injury, its clinical and mechanistic significance is increasingly evident (Ji et al., 2024).

## Short-chain fatty acids: molecular bridges between microbiota and host immunity

SCFAs are small organic acids produced by gut microbiota through the fermentation of indigestible dietary fibers (Riwes and Reddy, 2020). The primary SCFAs in the human gut are acetate, propionate, and butyrate, which are formed at an approximate molar ratio of 60:23:17 (Romick-Rosendale et al., 2018; Haller, 2018). The production of SCFAs occurs via bacterial fermentation processes that vary across different bacterial populations in the colon. These bacteria employ distinct pathways to ferment saccharides with intermediates, such as lactate, succinate, and ethanol, which are further converted by other bacterial taxa into SCFAs (Koh et al., 2016). For instance, the major propionate producers include various *Bacteroides* species and *Phascolarctobacterium succinatutens*, whereas butyrate is predominantly produced by species such as *Faecalibacterium prausnitzii* and *Eubacterium rectale* (Koh et al., 2016; Fusco et al., 2023).

Once produced, SCFAs are absorbed by colonocytes via passive diffusion and active transport via monocarboxylate transporters (Karim et al., 2024; Rekha et al., 2024; Dalile et al., 2019). SCFAs enter the bloodstream through the colonic epithelium and are involved in systemic effects, such as metabolic processes, brain function, and osteoblast differentiation (Fock and Parnova, 2023; Abdelhalim, 2024; Boets et al., 2017; Frampton et al., 2020; Kondo et al., 2022; Mann et al., 2024; Luu et al., 2018) (Figure 2).

SCFAs have emerged as critical mediators of the gut-immune axis, exerting extensive effects on both innate and adaptive immune responses (Corrêa-Oliveira et al., 2016). The role of SCFAs in modulating immune function is multifaceted and involves their influence on the differentiation and function of immune cells, maintenance of intestinal barrier integrity, and regulation of inflammatory processes (Luu et al., 2020; Gu et al., 2021). These effects are particularly significant in the context of HSCT, where SCFAs may substantially affect patient outcomes, including the development of GvHD (Li et al., 2022; Lin et al., 2021; Markey et al., 2020).

Among SCFAs, butyrate plays an indispensable role in preserving intestinal barrier integrity, a fundamental defense against luminal antigens and pathogens (Masetti et al., 2021; Schulthess et al., 2019). As the primary energy source for colonocytes, butyrate promotes epithelial proliferation and differentiation, essential processes for maintaining a robust intestinal barrier (Ji et al., 2024; Zhang et al., 2023a). Disruption of this barrier facilitates microbial translocation triggering systemic inflammation, a key driver of GvHD pathogenesis (Di Vincenzo et al., 2024; Schulthess et al., 2019; Jansen et al., 2022; Ghosh et al., 2020). SCFAs enhance the expression of tight junction proteins such as claudin-1, occludin, and ZO-1, reducing intestinal permeability and limiting bacterial translocation (Zhang et al., 2023b; Saleri et al., 2022). Furthermore, butyrate enhances mucus production, reinforcing the epithelial defense and preventing pathogen adhesion (Chen and Vitetta, 2020).

Beyond their localized effects, SCFAs exert systemic immunomodulatory effects by influencing both the innate and adaptive immune cells. SCFAs bind to specific receptors such as free fatty acid receptors (FFARs) and histone deacetylases (HDACs), which are expressed on immune cells, thereby modulating their differentiation, activation, and function. SCFAs modulate the activity of macrophages, neutrophils, and dendritic cells, which are primary players in the innate immune response (Mann et al., 2024). Butyrate, in particular, suppresses the production of pro-inflammatory cytokines by macrophages and promotes their differentiation toward an anti-inflammatory phenotype, which is essential for mitigating inflammatory conditions such as GvHD (Chang et al., 2014; Busnelli et al., 2019). Additionally, SCFAs influence neutrophil differentiation through FFAR2 activation, affecting migration and inflammasome activity, which are critical for inflammatory responses (Liu et al., 2023; Chun et al., 2019).

**Figure 3:**
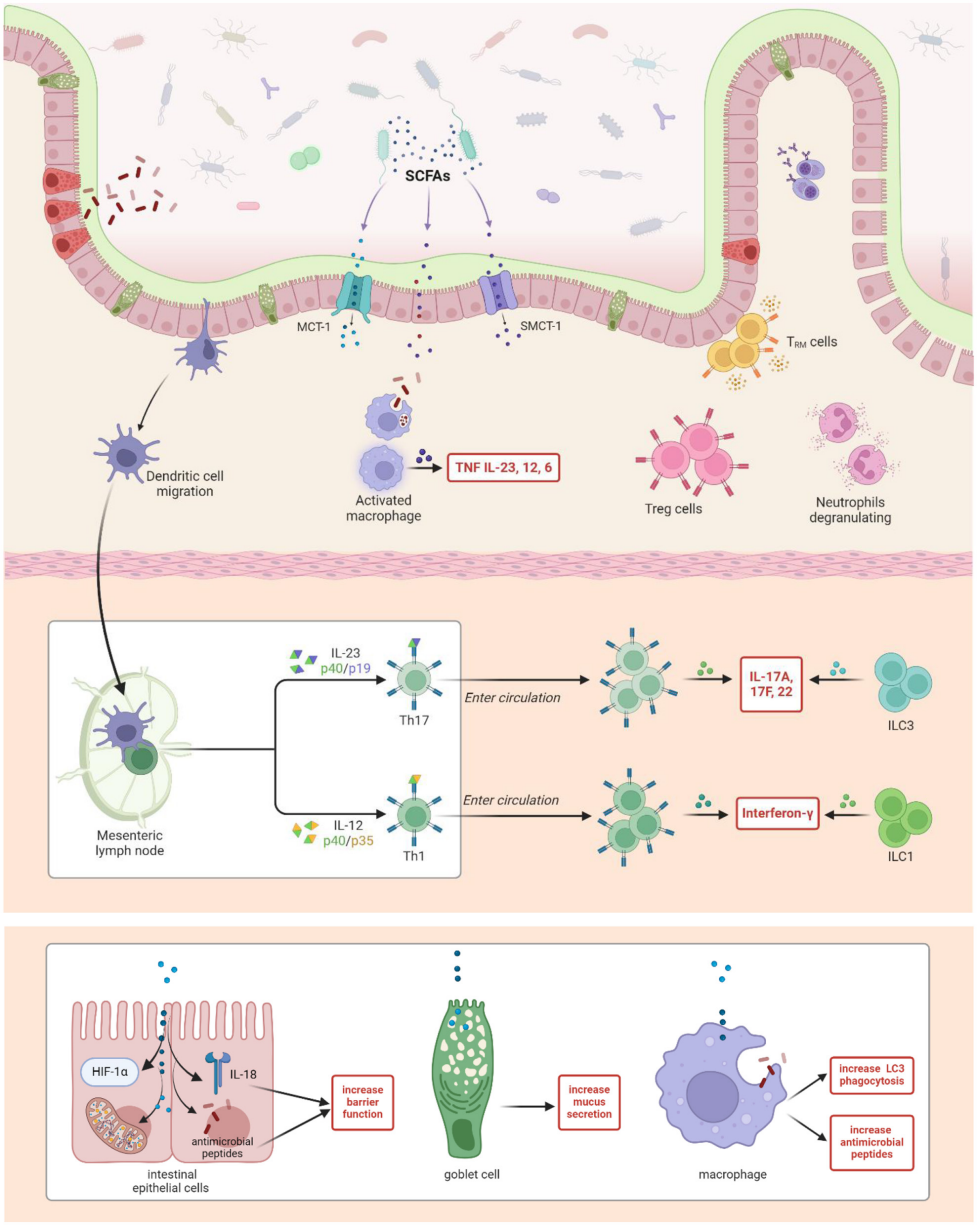
The role of short-chain fatty acids in modulating gut immunity. Short-Chain Fatty Acids (SCFAs) are absorbed by intestinal epithelial cells via specific transporters such as MCT-1 and SMCT-1, or through passive diffusion (Corrêa-Oliveira et al., 2016). SCFAs, particularly butyrate, enhance the integrity of the intestinal barrier by promoting the expression of tight junction proteins, stimulating mucus production by goblet cells, and increasing IL-18 and antimicrobial peptide production in intestinal epithelial cells (Corrêa-Oliveira et al., 2016; Schulthess et al., 2019; Wang et al., 2024). This results in a reinforced barrier that prevents the translocation of microbial products, thereby reducing inflammation and maintaining the gut homeostasis. SCFAs modulate the activity of various innate immune cells. They promote M2 polarization of macrophages, enhancing their anti-inflammatory and phagocytic functions, including the increased production of antimicrobial peptides and LC3-associated phagocytosis(Liu et al., 2023). SCFAs also influence neutrophil activity, including degranulation and inflammasome activation, thereby contributing to the regulation of inflammatory responses (Liu et al., 2023). Dendritic cell migration to mesenteric lymph nodes is depicted, where antigen presentation leads to T cell differentiation (Kim et al., 2014). SCFAs influence the differentiation of T cells into Th1 and Th17 cells through cytokine signaling (Liu et al., 2023; Kim, 2021). Th17 cells produce cytokines such as IL-17A, IL-17F, and IL-22, which further activate innate lymphoid cells (ILC3), whereas Th1 cells produce interferon-gamma (IFN-γ) and activate ILC1 cells (Park et al., 2015; Ma et al., 2019). Additionally, SCFAs promote the differentiation and function of regulatory T cells (Tregs), which are critical for maintaining immune tolerance and preventing excessive inflammatory responses (Liu et al., 2023). Together, these interactions demonstrate how SCFAs act as central mediators in gut immunity, linking microbial activity in the gut with host immune regulation, and maintaining the balance between immune defense and tolerance.

In adaptive immune system, butyrate promotes regulatory T cell (Treg) differentiation, which is essential for maintaining immune tolerance and suppressing excessive immune responses (Furusawa et al., 2013). SCFAs also inhibit the differentiation and function of pro-inflammatory T helper 1 (Th1) and Th17 cells, central players in GvHD pathogenesis (Schulthess et al., 2019; Park et al., 2015; Jin et al., 2022). Their role extends on B cells, where butyrate enhances Immunoglobulin A (IgA) production, a key element in mucosal immunity (Isobe et al., 2020) (Figure 3).

The anti-inflammatory properties of SCFAs are particularly relevant in GvHD, where inflammation exacerbates tissue damage and drives clinical manifestations. SCFAs downregulate the production of key pro-inflammatory cytokines such as tumor necrosis factor-alpha (TNF-α), interleukin-6 (IL-6), and interferon-gamma (IFN-γ), all of which are markedly elevated in GvHD (Fujiwara et al., 2018). Furthermore, SCFAs inhibit the activation of pivotal inflammatory signaling pathways, including the nuclear factor-kappa B (NF-κB) and mitogen-activated protein kinase (MAPK) pathways, which are implicated in GvHD pathogenesis (Kespohl et al., 2017).

Recent findings highlight the intricate interplay between SCFAs, bile acid metabolism, and the gut microbiome, revealing additional layers of immunoregulation with implications for GvHD. The gut microbiome mediates the conversion of primary bile acids produced by the liver into secondary bile acids (Houser and Tansey, 2017). SCFAs can modulate the composition and activity of bile acid-metabolizing bacteria, thereby altering the bile acid profiles within the gut, a factor that directly affects intestinal barrier integrity and immune regulation. Certain bile acids are known to reinforce the intestinal barrier, and SCFAs may modulate this relationship, potentially influencing susceptibility to GvHD (Malard et al., 2023b). Additionally, alterations in bile acid profiles driven by SCFAs can influence signaling pathways, such as the farnesoid X receptor (FXR), which plays a crucial role in inflammation and immune regulation (Riwes and Reddy, 2020; Masetti et al., 2021).

## SCFA-producing bacteria in HSCT: therapeutic potential and clinical strategies

HSCT induces significant shifts in the gut microbial landscape, particularly reducing the abundance and diversity of SCFA-producing bacteria (Peled et al., 2016; Zama et al., 2017). Studies consistently report a decline in *Clostridiales* species, especially those belonging to the *Lachnospiraceae* and *Ruminococcaceae* families, which are major butyrate producers central to gut homeostasis (Romick-Rosendale et al., 2018; Bansal et al., 2022; Skaarud et al., 2021). These alterations are driven by a combination of factors, including dietary restrictions, antibiotic exposure, and the cytotoxic effects of chemotherapy and radiotherapy on the intestinal niche.

The restrictive, low-fiber diets commonly prescribed during the peri-transplant period markedly reduce the availability of complex carbohydrates needed to sustain SCFA-producing commensals (Riwes et al., 2023; Andermann et al., 2021; D'Amico et al., 2019). In parallel, broad-spectrum antibiotic use for prophylaxis and infection management further exerts a profound and often prolonged impact on the microbiota. These agents indiscriminately deplete obligate anaerobes essential for SCFA synthesis, leading to lasting disruptions in microbial composition (Romick-Rosendale et al., 2018; Meedt et al., 2022; Shono et al., 2016). Romick-Rosendale et al. (2018) showed that increased antibiotic exposure correlated with a significant reduction in fecal butyrate and propionate levels during the first 14 days post-HSCT. Similarly, Shono et al. (2016) reported that treatment with imipenem-cilastatin and piperacillin-tazobactam was associated with greater microbiota damage and increased GvHD-related mortality, emphasizing the unintended consequences of antimicrobial regimens.

Beyond antibiotic-driven dysbiosis, conditioning regimens, particularly intensive chemotherapy and total body irradiation, inflict additional damage on the gut microbiome. These therapies compromise mucosal integrity, diminish microbial diversity, and facilitate the expansion of pathogenic taxa (Romick-Rosendale et al., 2018; Meedt et al., 2022). Ionizing radiation has been shown to severely reduce beneficial SCFA producers such as *Faecalibacterium* and *Bifidobacterium*, and deplete *Akkermansia muciniphila (A. muciniphila)*, a mucin-degrading bacterium crucial for maintaining epithelial homeostasis (Zhao et al., 2021; Fernandes et al., 2021).

Chemotherapy-induced microbiota alterations further exemplify the systemic consequences of gut dysbiosis in cancer therapy. In a longitudinal study of young adult cancer survivors, gut microbiota composition remained profoundly disrupted for up to six months post-chemotherapy, correlating with elevated markers of systemic inflammation and increased microbial translocation (Deleemans et al., 2019). These changes extended beyond immunological effects, contributing to neuropsychological symptoms resembling post-traumatic stress disorder (PTSD), including cognitive impairments such as memory deficits and executive dysfunction. Additionally, metabolic disturbances, including increased risk of obesity and metabolic syndrome, further underscored the long-term impact of chemotherapy on microbiome-mediated health outcomes.

In clinical and preclinical studies, abdominal irradiation led to significant losses of *A. muciniphila*, correlating with prolonged diarrhea in irradiated patients (He et al., 2023; Moraitis et al., 2023). Mechanistic work has demonstrated that *A. muciniphila* promotes gut barrier integrity through propionate-mediated activation of GPR43, which enhances expression of tight junction proteins including occludin and ZO-1 (He et al., 2023).

Microbiome disruptions in HSCT patients also profoundly affect SCFA production and metabolism, leading to cascading effects on immune function, epithelial barrier repair, and systemic inflammation. Intestinal inflammation and mucosal injury impair SCFA absorption by colonocytes, while shifts in microbial composition alter the relative ratios and types of SCFAs produced, potentially influencing their downstream immunometabolic effects (Riwes and Reddy, 2020; Romick-Rosendale et al., 2018; Sen and Thummer, 2022; Ghimire et al., 2021; Mathewson et al., 2016; Tan et al., 2014). Given that barrier dysfunction is a well-established contributor to GvHD pathogenesis, strategies aimed at restoring SCFA availability and supporting epithelial repair hold strong therapeutic potential (Riwes and Reddy, 2020; Ji et al., 2024).

By dampening inflammatory signaling and promoting tissue restitution, SCFAs emerge as key regulators of host-microbiota equilibrium with implications for GvHD modulation. Clinical studies support this role: in one cohort, reduced butyrate and propionate levels were associated with an increased incidence of chronic GvHD (Markey et al., 2020). Prolonged depletion of butyrate-producing bacteria during the early post-transplant period also correlated with greater gastrointestinal GvHD severity and increased transplant-related mortality (Meedt et al., 2022). These findings highlight the importance of preserving or restoring SCFA producers to mitigate post-transplant complications.

Emerging strategies to enhance microbiome resilience and SCFA recovery include targeted dietary interventions, probiotic administration, and microbial therapeutics. In murine models, supplementation with *Bacteroides fragilis* improved gut barrier integrity, increased Treg responses, and reduced GvHD severity without compromising graft-vs.-leukemia effects (Sofi et al., 2021). In clinical settings, supplementation with resistant potato starch significantly elevated fecal butyrate levels, offering a dietary route to bolster SCFA-mediated immune modulation (Riwes et al., 2023).

Preclinical studies have further demonstrated that high-fiber diets enrich SCFA producers such as *Bacteroides acidifaciens*, improving outcomes after radiotherapy and enhancing antitumor immunity (Then et al., 2020). Mice with elevated SCFA levels following irradiation showed improved epithelial regeneration, increased T-cell activation, and reduced treatment-related toxicity. In parallel, probiotics have shown protective effects against radiation-induced neuroinflammation and cognitive decline (Venkidesh et al., 2023). Beyond local effects, SCFAs modulate systemic immunity, enhance chemotherapy sensitivity, and regulate inflammation. Butyrate, acetate, and propionate have demonstrated synergistic anti-tumor properties, particularly in colorectal cancer, by promoting cytotoxic T-cell function and modulating epigenetic pathways (Al-Qadami et al., 2022).

Altogether, these findings underscore the therapeutic promise of restoring SCFA-producing communities in HSCT recipients. Understanding and leveraging these microbiota-derived metabolites may represent a pivotal step in designing microbiome-based strategies to enhance immune recovery, reduce transplant-related morbidity, and improve long-term outcomes. The main findings of studies investigating SCFA roles post-HSCT are summarized in Tables 1, 2.

**Table 1:** Summary of key findings from observational studies investigating the role of SCFAs in post-HSCT patients.

**Authors**	**Country**	**Study design**	**Population**	**Main findings**	**Limitations**
Artacho et al. (2024)	Spain	Prospective, observational, single-site, multi-omics	Adult allo-HSCT recipients with hematologic malignancies	- Allo-HSCT altered the microbiome, depleting *Clostridiales* and reducing acetate and malonate. - Expansion of *Staphylococcus* spp. linked to GvHD and infections. - Specific antibiotics modulated these microbial shifts.	- No validation cohort. - Small sample size. - Associations not confirmed in experimental models.
Thiele Orberg et al. (2024)	Germany	Prospective, longitudinal, multi-site observational study.	Allo-HSCT recipients, varied malignancies, GI-GvHD severity levels.	- Microbiome signature with *Lachnospiraceae, Oscillospiraceae*, and bacteriophages correlated with protective SCFA and immunomodulatory metabolites. - Sustained metabolite production improved survival and reduced mortality. - FMT rescued microbiome depletion and resolved steroid-refractory GVHD.	- Challenges in gut virome analysis due to high viral diversity. - Limited reference databases for viral binning. - Single-case FMT treatment requires larger validation.
Bansal et al. (2022)	USA	Prospective observational longitudinal study.	Auto and allo-HSCT recipients.	- Antibiotics are the primary driver of microbiome diversity loss post-HSCT, with recovery by day +100. - SCFA-producing bacteria (*Ruminococcaceae, Blautia*) decline in both transplant groups. - Severe acute GvHD (grade II-IV) is linked to a greater loss of SCFA producers (*Ruminococcaceae, Eubacterium dolichum, Bifidobacterium*) and an increase in *Bacteroides ovatus*, associated with inflammation. - Pre-transplant microbiome protection may improve outcomes.	- Retrospective nature. - Small sample size. - Limited subgroup analysis and manual annotation.
Meedt et al. (2022)		Prospective single-center observational.	Adult HSCT patients, healthy donors.	- Prolonged suppression of butyrate-producing bacteria post-HSCT. - Early broad-spectrum antibiotic use linked to lower butyrate levels. - Lower butyrate levels associated with severe GI-GvHD and higher mortality.	- Not randomized. - Did not control for host variables like geography, diet, or BMI. - Larger, multicenter validation needed.
Schwabkey et al. (2022)		Single-site observational study, retrospective and prospective murine interventions	Adult HSCT recipients, neutropenic post-treatment.	- Post-HSCT fever is associated with increased *Akkermansia* and *Bacteroides*. - Radiotherapy or melphalan elevates *A. muciniphila* in mice, likely due to reduced dietary intake. - Azithromycin lowers *Akkermansia* and mitigates related complications. - Caloric restriction increases *A. muciniphila* and thins colonic mucus, an effect reversed by antibiotics. - It also reduces SCFAs (acetate, propionate, butyrate) while raising succinate. - Increased acidity and propionate inhibit *A. muciniphila* growth and mucin degradation.	- High variation in *A. muciniphila* abundance. - Unclear mechanism of propionate suppression on mucin utilization. - Functional strain differences not explored
Burgos da Silva et al. (2022)	USA	Observational, retrospective, single-site	Adult allo-HSCT recipients with and without GvHD.	- Microbiome preservation linked to reduced GvHD severity. - Lower Clostridia and butyrate producers associated with worse outcomes. - Pre-GvHD microbiome markers linked to survival improvement.	- Observational, retrospective nature prevents causality establishment. - Antibiotic use impact confounds results. - High microbiome heterogeneity complicates analysis.
Ghimire et al. (2021)		Observational, stratified design	Adults undergoing allo-HSCT.	- SCFA receptors GPR109A and GPR43 are upregulated in severe acute GvHD. - Broad-spectrum antibiotics suppress GPR and FOXP3 expression, disrupting commensal bacterial protection. - GPR43 expression correlates with NLRP3 inflammasome activation, but only in antibiotic-free patients. - *In vitro*, SCFAs (especially butyrate) increase GPR109A and GPR43 in monocyte-derived dendritic cells, shifting cytokine balance toward anti-inflammatory IL-10 and reducing pro-inflammatory IL-12. - Antibiotic-induced loss of GPR and FOXP3 expression highlights the protective role of the commensal-SCFA-GPR axis.	- Unable to directly assess microbiome status at biopsy retrieval. - Used antibiotic treatment as a microbiome status surrogate. - Unclear role of translocated bacteria and tissue metabolites.
Markey et al. (2020)	USA, Germany	Case-control, cross-sectional, multi-site, observational	Allo-HSCT recipients at risk of or experiencing cGvHD.	- Low butyrate and propionate linked to cGvHD development. - Butyrate-producing bacteria (*Lachnoclostridium, Faecalibacterium*) reduce cGVHD risk. - Gut microbiome exerts immunomodulatory effects post allo-HSCT.	- Preliminary findings need further validation. - Treatment and demographic differences affect model accuracy. - Generalizability limited due to case-control
D'Amico et al. (2019)		Observational, longitudinal, pediatric HSCT study	Pediatric HSCT patients.	- Enteral nutrition (EN) restores gut microbiome homeostasis post-HSCT. - EN reduces bloodstream infection risk and promotes SCFA recovery.	- Conflicting results in adult HSCT studies.
Haak et al. (2018)	USA	Prospective observational study, single-site	Adults undergoing allo-HSCT (leukemia patients, umbilical cord transplant recipients).	- Higher butyrate-producing bacteria abundance reduces viral LRTI risk 5-fold. - High butyrate bacteria abundance predicts protection against viral LRTI.	- Broad LRTI definition may cause misclassification. - Uncertainty if protection is conferred by bacteria or metabolites. - Data collected only at engraftment, not later time points.
Romick-Rosendale et al. (2018)	USA	Prospective observational, single-site	Children undergoing HSCT.	- Progressive declines in fecal SCFAs, particularly butyrate and propionate, post-HSCT. - High antibiotic exposure associated with reduced SCFA levels. - Lower SCFAs correlated with increased GvHD incidence.	- Lower-than-average GvHD incidence in study group. - Differences between human and murine models. - Need for clinical trials to validate findings.
Shono et al. (2016)	USA	Retrospective observational cohort, murine model	Adults undergoing allo-HSCT for hematologic malignancies.	- Imipenem-cilastatin and piperacillin-tazobactam are linked to increased GvHD-related mortality in allo-HSCT recipients. - Aztreonam and cefepime showed no such association. - Murine models confirm that imipenem-cilastatin and piperacillin-tazobactam worsen GvHD severity. - Despite differences in *Clostridiales* abundance (major SCFA producers), SCFA levels remained unchanged between patients treated with aztreonam or imipenem-cilastatin.	- Retrospective single-center study. - Association but not causation established. - Needs validation by prospective trials.

**Table 2:** Summary of key findings from Interventional studies investigating the role of SCFAs in post-HSCT patients.

**Authors**	**Country**	**Study design**	**Population**	**Main findings**	**Limitations**
DeFilipp et al. (2024)		Open-label, single-arm, pilot study.	Adults with high-risk acute GvHD post allo-HSCT.	- FMT led to expansion of donor-derived bacterial species and increased tryptophan metabolites and SCFAs within 7 days. - Complete responders showed distinct stool metabolite shifts, including higher levels of 5-HIAA, indole, indoxyl sulfate, serotonin, and SCFAs (butyric acid, valeric acid, isobutyric acid, isovaleric acid). - 9/10 participants completed all FMT doses. - 70% complete response rate for lower GI GvHD by day 28.	- Small sample size. - Single-arm study. - Concurrent FMT and corticosteroids limit.
Bu et al. (2024)		Randomized, controlled, repeated measures.	Mice and human peripheral blood donors.	- Human amniotic mesenchymal stem cells (hAMSCs) prevent aGvHD by repairing the intestinal barrier and improving microbiome dysbiosis in a microbiome-dependent manner. - aGvHD reduces SCFA concentrations (propionate, butyrate, valerate), while hAMSCs significantly restore SCFA levels, with butyrate increasing tenfold. - SCFA elevation (propionate, butyrate, isobutyrate, valerate, isovalerate) correlates with tight junction protein expression (ZO-1, occludin), supporting intestinal barrier integrity.	- Small sample size. - Different samples used for sequencing and metabolomics, preventing correlation analysis. - Further investigation needed into SCFAs' role.
(Malard et al., 2023b)	France	Prospective, single-arm, open-label, multicenter (26 sites).	Adults with steroid-resistant aGvHD post allo-HSCT.	- Pooled allogeneic fecal microbiota MaaT013 showed a 38% GI-response rate at day 28 (HERACLES study) and 58% response in expanded access program. - Standardized pooled allogeneic FMT increased bacterial diversity and abundance of beneficial bacteria such as butyrate-producing bacteria. - No definitive link between FMT and infections.	- No prior prospective studies on FMT in GI-GvHD. - Single-arm, non-randomized design. - No formal sample size calculation.
Riwes et al. (2023)	USA	Single-center, prospective, single-arm, longitudinal.	Adults undergoing myeloablative allo-HSCT.	- Resistant potato starch (RPS) is feasible, safe, and well tolerated post-HSCT. - Fecal butyrate levels increased with RPS administration. - Intestinal and plasma metabolites were significantly altered.	- Small sample size. - Relied on data from healthy cohorts. - Differences in donor sources between RPS and control groups.
Skaarud et al. (2021)		Randomized controlled trial (RCT), open, two-armed	Adults undergoing allo-HSCT for hematologic malignancies..	- Nutritional intervention had no significant effect on microbiota composition, SCFAs, or gut barrier markers. - Low microbial diversity at 3 weeks post-HSCT correlated with higher one-year mortality. - SCFA levels declined significantly in both intervention and control groups, indicating allo-HSCT negatively impacts gut microbiota. - Higher baseline fecal propionic acid, valeric acid, and total SCFAs were linked to improved overall survival and lower non-relapse mortality. - SCFA changes did not differ between intervention and control groups, suggesting the dietary strategy was insufficient to prevent microbiota disruption post-HSCT.	- Small sample size. - Not designed to compare nutrition support routes. - Potential bias due to disease severity and treatment differences.
Andermann et al. (2021)		Phase I pilot, single-arm, dose-escalation, single-site.	Adults undergoing reduced-intensity allo-HSCT with hematologic malignancies.	- Fructooligosaccharide (FOS) at 10 g/d was well-tolerated in allo-HSCT patients without significant adverse effects. - Gut microbiota composition differed between FOS and control groups on transplant day, but these changes did not persist post-transplant. - No significant impact on gut metabolic pathways, SCFA levels, or peripheral Tregs, though FOS showed a trend toward higher Tregs and increased CD4+ T cell activation marker (CTLA4+).	- Single-center, small sample size. - FOS intake inconsistent due to mucositis. - Short duration and lack of symbiotic.

## Microbial metabolites and engineered therapies in HSCT: discussion and future perspectives

Short-chain fatty acids (SCFAs), particularly butyrate, have emerged as critical mediators of intestinal and systemic immune homeostasis, with profound implications for hematopoietic stem cell transplantation (HSCT) outcomes (Romick-Rosendale et al., 2018; Riwes et al., 2023; D'Amico et al., 2019; Meedt et al., 2022). Acting at the intersection of microbiome-host crosstalk, butyrate modulates inflammatory pathways, epithelial repair, and epigenetic programming. Its multifaceted roles in shaping immune reconstitution and barrier function render it a promising candidate for therapeutic exploitation. However, translating these microbiota-derived metabolites into clinical interventions demands a transition from descriptive associations toward a mechanistic, causality-based framework.

Despite compelling evidence for SCFA-mediated immune modulation, the precise mechanistic pathways remain incompletely understood (Zhao et al., 2025). Current studies inconsistently implicate GPR43, GPR41, or GPR109A as dominant receptors; the relative contribution of each remains unclear due to divergent findings in knockout models and overlapping ligand specificity (Muralitharan et al., 2023; Behler-Janbeck et al., 2024). Moreover, context-specific responses, shaped by the tissue microenvironment, immune status, and epithelial-immune crosstalk, are not consistently delineated, making it difficult to predict therapeutic efficacy across varied clinical settings (Zhao et al., 2025; Dai et al., 2025). Most models further isolate SCFAs from the broader “metabolomic milieu”, neglecting complex interactions with microbial metabolites such as bile acids or tryptophan derivatives (Chulenbayeva et al., 2025). This reductionist approach may mask synergistic or antagonistic effects, thereby limiting clinical translatability (Chulenbayeva et al., 2025).

The therapeutic potential of SCFAs is rooted in their ability to exert potent anti-inflammatory effects by inducing regulatory T cells (Tregs) and inhibiting histone deacetylases (HDACs), thus orchestrating gene expression across immune and epithelial compartments (Boets et al., 2017; Fujiwara et al., 2018; Kespohl et al., 2017). Beyond the gut, recent findings reveal that SCFAs influence hematopoietic recovery by regulating the epigenetic landscapes of progenitors, impacting cell fate through chromatin remodeling and metabolic rewiring (Ji et al., 2024; Peled et al., 2016; Park et al., 2015; Chi et al., 2023; Nshanian et al., 2025; Kopczyńska and Kowalczyk, 2024). Notably, butyrate enhances chromatin accessibility via histone acetylation to promote epithelial lineage specification, a paradigm directly relevant to mitigating epithelial injury post-conditioning (Stein and Riber, 2023).

However, the role of butyrate in HSCT may be biphasic and context-dependent, acting as a “double-edged sword” depending on the structural integrity of the intestinal crypts. While butyrate is protective during steady-state or preventive phases, it may be detrimental during active mucosal injury. Mechanistically, Kaiko et al. (2016) demonstrated that while differentiated colonocytes utilize butyrate as a primary energy source, colonic stem/progenitor cells are susceptible to its growth-inhibitory effects via a Foxo3-dependent pathway. In the healthy gut, the crypt architecture acts as a “metabolic barrier,” where differentiated colonocytes at the crypt surface metabolize luminal butyrate, preventing it from reaching the stem cell niche at the base (Kaiko et al., 2016). In the context of HSCT, the profound mucosal denudation caused by conditioning or acute GvHD likely breaches this shield, exposing stem cells to inhibitory butyrate concentrations and thereby delaying epithelial repair. This mechanistic rationale is reinforced by clinical observations from Golob et al. (2019) which linked a higher abundance of butyrogenic bacteria following the onset of acute gastrointestinal GvHD with the development of steroid-refractory and chronic GvHD. These findings suggest that the therapeutic window for SCFA interventions must be carefully calibrated; butyrate-producing consortia may reinforce the barrier early in the transplant course but could potentially hinder recovery if present during windows of severe epithelial denudation.

To address these complexities and the limitations of traditional probiotics, which suffer from poor engraftment or safety concerns in immunocompromised hosts (Yazdandoust et al., 2023; Ma et al., 2023; Lee et al., 2023), the field is shifting toward “living therapeutics” and precision delivery. Synthetic biology has introduced a new frontier: programmed *Saccharomyces cerevisiae* (Wu et al., 2023) and *E. coli* (Jin et al., 2025) chassis designed to detect inflammatory cues and release butyrate or postbiotics in a controlled, spatiotemporal manner. Such engineered systems could potentially personalize metabolite release to avoid “toxic” windows while maximizing local mucosal repair. Parallel to these efforts, the development of butyrate-loaded nanocarriers and pH-sensitive capsules is essential to ensure targeted colonic delivery, overcoming bioavailability issues of dietary fiber (Lopes et al., 2023).

Crucially, the immunomodulatory role of the microbiome extends beyond a single metabolite class. Tryptophan catabolites activate the aryl hydrocarbon receptor (AhR) to promote mucosal tolerance, while secondary bile acids influence T cell polarization (Michonneau et al., 2019; Landfried et al., 2011; Swimm et al., 2018; Tyszka et al., 2024; Haring et al., 2021). Emerging data suggest a “metabolic complementarity,” where SCFAs may synergize with tryptophan and bile acid pathways to reinforce the immunological barrier post-HSCT (Cao et al., 2024). Failure to account for these interactions, alongside cross-species discrepancies between murine and human models, remains a significant hurdle in developing regulatory-grade therapeutics.

Future research must prioritize causal inference through harmonized, precision-driven trial designs. Patient stratification should move beyond clinical GvHD grading to include validated biomarkers of epithelial injury and longitudinal metabolomic profiling to identify the optimal “metabolic window” for intervention. Trials must standardize delivery routes, dosing, and colonic release profiles while rigorously adjusting for confounders such as corticosteroids and antimicrobials. Ultimately, the microbiome is a dynamic metabolic organ. Realizing its promise will require a convergence of precision nutrition, synthetic biology, and patient-specific profiling to unlock a new era of transplant medicine, one where engineered microbial bioactivity is precisely timed to support immune reconstitution and survival.
